# Automated identification of crystallographic ligands using sparse-density representations

**DOI:** 10.1107/S1399004714008578

**Published:** 2014-06-29

**Authors:** C. G. Carolan, V. S. Lamzin

**Affiliations:** a European Molecular Biology Laboratory (EMBL), c/o DESY, Notkestrasse 85, 22603 Hamburg, Germany

**Keywords:** macromolecular X-ray crystallography, ligands, drug design, shape descriptors

## Abstract

A novel procedure for the automatic identification of ligands in macromolecular crystallographic electron-density maps is introduced. It is based on the sparse parameterization of density clusters and the matching of the pseudo-atomic grids thus created to conformationally variant ligands using mathematical descriptors of molecular shape, size and topology. In large-scale tests on experimental data derived from the Protein Data Bank, the procedure could quickly identify the deposited ligand within the top-ranked compounds from a database of candidates. This indicates the suitability of the method for the identification of binding entities in fragment-based drug screening and in model completion in macromolecular structure determination.

## Abstract 2

A novel procedure for identifying ligands in macromolecular crystallographic electron-density maps is introduced. Density clusters in such maps can be rapidly attributed to one of 82 different ligands in an automated manner.

## 1. Introduction   

Ligand molecules are present in many macromolecular crystals and frequently indicate the function of the parent protein or nucleic acid. Ligand identification and the elucidation of ligand-binding modes in the structures derived from these crystals underpins efforts to assess the macromolecule’s mechanism of action and potential means by which these actions can be manipulated (Abendroth *et al.*, 2011 ▶; Li *et al.*, 2005 ▶). In classic structure-based drug design, where a specific lead or drug compound has been added to the system prior to (*e.g.* co-crystallization) or subsequent to (*e.g.* soaking) crystallization experiments, identification of the ligands giving rise to difference electron density following macromolecular model building is generally facile. However, it is less straightforward when small molecules, typically endogenous substrates or effectors that adhere to the protein during expression, remain bound during purification and crystallization (Hamiaux *et al.*, 2009 ▶; Girardi *et al.*, 2010 ▶) or when multiple ligands are added to crystals simultaneously. The latter approach may improve efficiency in fragment-based drug design (Mooij *et al.*, 2006 ▶) and in metabolite cocktail screening for identification of protein function (Shumilin *et al.*, 2012 ▶). In macromolecular crystallography (MX), small-molecule entities are also derived from crystallization media or cryoprotectant solutions, and the identification and fitting of these into electron-density maps is necessary in order to explain the experiment more fully. Bearing in mind that the PDB ligand database (Golovin *et al.*, 2004 ▶) now contains 17 000 entries, the task is clearly not trivial. Frequent discussions on the nature of electron-density ‘blobs’ as well as the often-questionable assignment of ligand structures to such blobs (Kleywegt, 2007 ▶) attest to the complexity of a task that has hitherto relied on the expertise of the researchers involved and their subjective analyses. Evidently, an automated and efficient approach to the unbiased and accurate identification of ligands in electron-density maps is desirable.

A variety of methods for the automated fitting of known ligands into electron-density maps have been proposed, typically based either on the recognition of the location of a rigid core of a ligand in electron density prior to full addition of other elements of the ligand (Oldfield, 2001 ▶; Terwilliger *et al.*, 2006 ▶), the alignment of the ligand with the principal axes of the density and fitting using Metropolis-type optimization (Debreczeni & Emsley, 2012 ▶), or a combination of similar methods (Evrard *et al.*, 2007 ▶; Langer *et al.*, 2012 ▶). Clearly, these methods can be adapted for ligand identification by writing scripts to cyclically fit each ligand from a database of molecules to a specified electron-density cluster. Indeed, Terwilliger *et al.* (2007 ▶) demonstrated the usefulness of such an approach, ranking each of the models produced by their ligand-modelling protocol for a database of 119 ligands by electron-density map correlations, and noting that the correct entity (*i.e.* that deposited in the PDB entries used in testing) was also the top-ranked compound in 46% of cases. However, such an approach is inherently slow, since it necessitates the fitting of all candidate ligands to the density.

Efforts to more rapidly match ligands to the electron density have focused on the use of mathematical descriptors, as comparison of their values can be both quick and robust. Even simple shape features such as the volume of the bounding box of a ligand molecule or density cluster can be used to identify the appropriate density blob in a difference map prior to ligand structure modelling (Langer *et al.*, 2012 ▶). The more challenging task of identifying a conformationally variable ligand from its density given a large database of candidates obviously requires methods of higher sophistication. Gunasekaran *et al.* (2009 ▶) used three-dimensional Zernike moments to match ligands to the segmented electron-density clusters obtained from OMIT maps, but despite the high level of rigour associated with such an approach, the correct ligand was identified at the top of the ranking in only 30% of cases.

An interesting approach to modelling ligand electron density used a graph representation of the central axis of a density cluster (Aishima *et al.*, 2005 ▶) with subsequent structure modelling using geometrical and conformational matching of the ligand to the graph. The advantage of representing density as a point graph has already been emphasized by the use of an atomic labelling algorithm to match ligand atoms to the free atoms of a sparse grid built within the electron-density blob (Zwart *et al.*, 2004 ▶). By representing electron density in a pseudo-atomic manner, it becomes possible to use features based on interatomic distances and connectivities to describe both the density and the candidate ligands.

In this manuscript, we present a novel and effective method for the fast parameterization of ligand electron density as a pseudo-atomic point cloud and introduce the application of a variety of mathematical features that describe molecular size, shape and topology to enable the efficient matching of ligand candidates to electron density. The methodology can rapidly yet accurately identify ligands in experimental macromolecular crystallographic density maps and is expected to be useful as both a modelling and a validation tool.

## 2. Methods   

### 2.1. An overview of the method   

**Figure 1:**
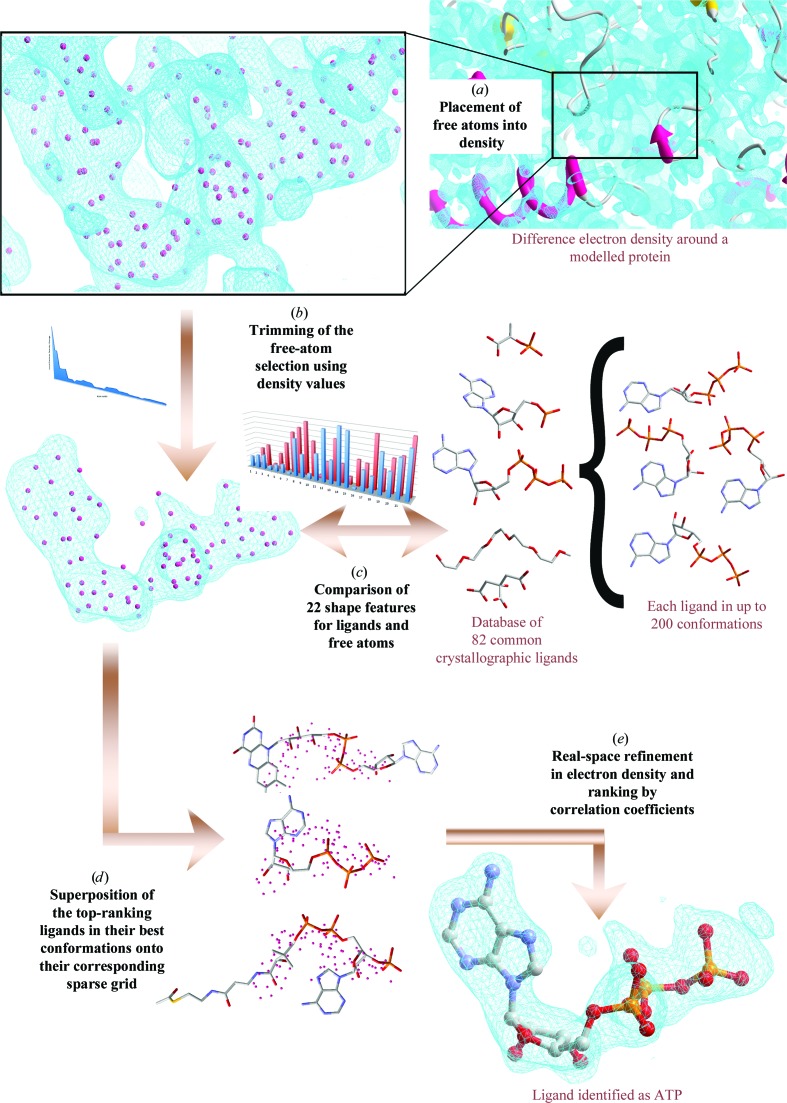
Schematic representation of the protocol for ligand identification, shown for adenosine triphosphate (ATP) in the structure of a putative N-type ATP pyrophosphatase (PDB entry 3rk1; Forouhar *et al.*, 2011 ▶) at 2.3 Å resolution. The (*F*_o_ − *F*_c_, α_c_) difference density map is shown contoured at 1.0σ above the mean; free atoms are shown as balls. The thickness of the visual slab has been adjusted for each image to provide the best view; however, it is reduced in (*b*) in order to clarify the electron density of interest following protein model display in (*a*).

The method for screening a database of candidate ligand compounds is delineated in Fig. 1 ▶. Specifically, free atoms are used to parameterize a specified electron density and a series of mathematical features are calculated based on the locations of the generated sparse-density points. These are compared with the same features calculated for each conformation of the candidate ligands and a ranking is deduced based on the weighted sum of the scores for each feature. The highest-ranking compounds, each in turn in their top-scored conformations, are subjected to brief real-space refinement in the electron-density map. Final rankings are based on the correlation coefficient between the refined ligands and the electron density.

### 2.2. Selection of unique ligands   

We created a large data set of ligands commonly found in crystal structures, containing both endogenous ligands and compounds derived from the experimental procedures common to MX. Analysis of the Protein Data Bank (PDB; Berman *et al.*, 2000 ▶) in May 2013 indicated that there were over 15 000 different ligand entities in total. 294 of these were present in at least 40 different deposited structures and were therefore regarded as being common. As our focus of interest was on noncovalently bound ligands that typically give rise to isolated blobs in MX electron-density maps, modified amino acids such as phosphoserine (SEP) and *O*-sulfo-l-tyrosine (TYS) as well as saccharides involved in post-translational glycosylation were not considered. Ligand entities with less than five non-H atoms (mainly single-atom ions) were also excluded.

Closer inspection of the remaining 140 ligands highlighted the fact that many of them are very similar to each other. For example, ligands such as adenosine-5′-triphosphate (ATP), phosphomethylphosphonic acid-adenylate ester (ACP) and phosphoaminophosphonic acid-adenylate ester (ANP) have identical substructures (with respect to their non-H atoms) and differ only in atomic makeup. In place of the O atom between the second and third phosphates in ATP, ACP has a single C atom, while ANP has an N atom. Recognition of these ligands from their electron density is only possible in maps of very high resolution where atomic identity or hydrogen-bonding networks can be identified, precluding their differentiation by the methods described herein. Therefore, the ligands were clustered to reduce such substructural redundancy. Descriptors based on interatomic bonding patterns in small molecules, such as the widely used BCUT descriptors (the eigenvalues of symmetric matrices in which the terms represent bonds and bond orders between atoms; Burden, 1989 ▶, 1997 ▶), are conformationally invariant and very suitable for such a clustering task. Such features were calculated for each of the ligands being considered. *k*-means clustering followed by manual curation of the results yielded 82 unique ligands ranging in size from five (sulfate, SO_4_, and imidazole, IMD) to 100 non-H atoms (cardiolipin, CDL) in groups of up to eight different ligands.

### 2.3. Selection of training and test data sets   

Experimental structures and structure factors for all entries in the PDB containing at least one of the 82 ligands in the ligand test set were downloaded. Only structures derived using X-ray crystallography with resolutions between 1.0 and 2.5 Å and present in the Electron Density Server (EDS; Kleywegt *et al.*, 2004 ▶) were used. MTZ datafiles were prepared using the *CIF*2*MTZ* program from the *CCP*4 package (Winn *et al.*, 2011 ▶).

In order to reduce the ‘memory’ of the deposited ligand structure in the density map, re-refinement of the ‘apo’ protein was undertaken. Specifically, all ligand and solvent atoms were removed from the PDB files and restrained refinement of the protein against the X-ray data was executed using *REFMAC* (Murshudov *et al.*, 2011 ▶).

The 5025 PDB entries thus obtained were further filtered based on the correlation coefficient between the maps calculated from the deposited ligand structure and the difference maps obtained after *REFMAC* refinement, with a threshold of 0.75 being applied; correlation coefficients were calculated using the *CCP*4 programs *SFALL* and *OVERLAPMAP* [see, for example, Muller (2013 ▶) or Pozharski *et al.* (2013 ▶) for a discussion on thresholds for correlation coefficients]. This resulted in elimination of two thirds of the entries, highlighting the rather poor-quality and inadequate interpretation of ligand electron density in many PDB cases that has been noted in several reports (Kleywegt, 2007 ▶; Cooper *et al.*, 2011 ▶; Liebeschuetz *et al.*, 2012 ▶; Pozharski *et al.*, 2013 ▶) and presents obvious difficulties for model building and validation. Nonetheless, more than 1100 different PDB entries were available for use. 160 of these were placed into a ‘training set’ for refinement of the method, while the remaining 970 entries were used for the evaluation described later.

### 2.4. Parameterizing electron density   

Difference electron density is computed at 0.3 Å spacing and the user provides the approximate location of the density cluster of interest. Preparation of the final grid for shape comparison proceeds as follows.

**Figure 2:**
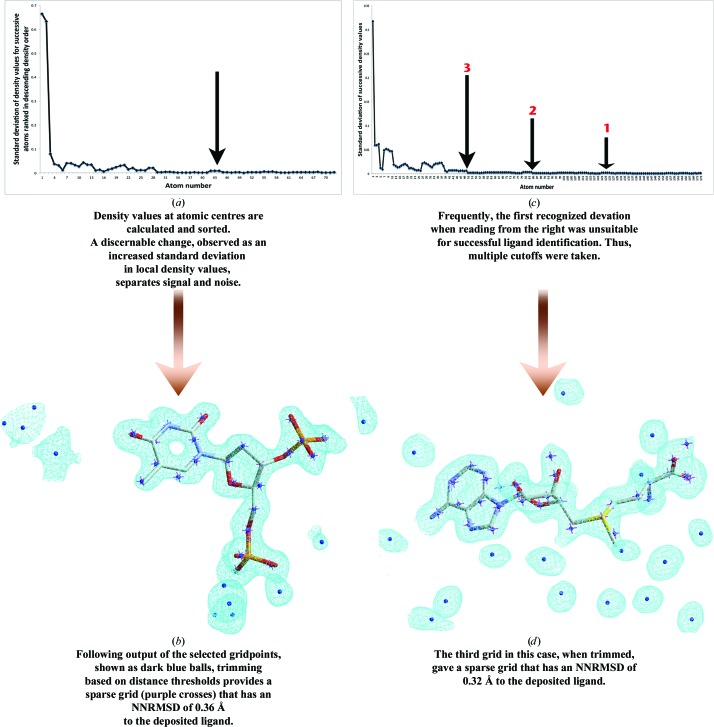
Trimming of pseudo-atomic grid clusters for feature comparison with ligand features. Difference (*F*_o_ − *F*_c_, α_c_) maps are shown contoured at 2.5σ above the mean. (*a*) Density values for placed free atoms are sorted in descending order and the differences in adjacent values are calculated. The standard deviations of density differences are plotted, and only those atoms with density higher than the marked point are output. The data are shown for PDB entry 4iun (Li *et al.*, 2010 ▶). (*b*) The output atoms, shown as balls, are trimmed further based on distance cutoffs to produce the final shape for screening, shown as crosses. It is an excellent match to the deposited ligand, THP. (*c*) As in (*a*) but with three clusters identified for the data in PDB deposition 3mb5 (Guelorget *et al.*, 2010 ▶) are marked with arrows. (*d*) The third cluster, marked by arrow 3, is a good match to the final ligand, SAM.

(i) The density is parameterized by placing free atoms onto a grid biased towards grid points with higher density. Every free atom has a neighbour at between 1.2 and 1.7 Å distance.
(ii) A threshold of 2.0 Å distance is applied between free atoms to select the cluster closest to the point of interest. All other free atoms are removed.
(iii) Electron-density values at the locations of each free atom are obtained and sorted in descending order. The number of free atoms in the selected cluster is typically less than 200. For each density value the standard deviation (σ) of the density-value differences amongst successively sorted atoms is calculated; for example, σ for atom *i* is calculated from the three successive differences in density values between atoms *i* − 3, *i* − 2, *i* − 1 and *i*. Since the density values are sorted, the differences between their successive values are higher at the edges of the density cluster and this is reflected by peaks in σ values, as depicted in Fig. 2 ▶(*a*). A threshold is set at the position of the peak and up to five different thresholds are used to thin the sparse grids (Fig. 2 ▶ *a*).
(iv) Following grid thinning, step (ii) is repeated with the maximum inter­atomic distance threshold increased to 2.3 Å, producing the final molecular shape(s) for comparison using mathematical features (Fig. 1 ▶).

Grid thinning and clustering takes crystallographic symmetry into account so that ligands located across formal borders between different asymmetric units can be properly recognized.

### 2.5. The numerical feature descriptors   

Previous experiences working with shape and topological features to model molecular fragments into crystallographic electron density highlighted a range of such features that can be used (Langer *et al.*, 2012 ▶; Hattne & Lamzin, 2008 ▶, 2011 ▶; Heuser *et al.*, 2009 ▶).

A total of 22 features were selected for use and are enumerated in Table 1 ▶. They are all invariant with respect to translation and rotation of the ligand, and all except the number of atoms are invariant to the ligand size. Only the eigenvalues of the connectivity matrices (Burden, 1989 ▶, 1997 ▶) and, of course, the number of atoms are conformation-invariant; the others are dependent.

The features were pre-computed for all conformations (up to 200) of each of the 82 ligands: a total of about 10 000 entries. Shape comparisons are carried out against the sparse grids in the density cluster at all (up to five) σ thresholds. Pseudo-connectivity between free atoms was derived as described in Langer *et al.* (2012 ▶). Ranking is based on a composite of all scores for all ligands against all sparse grids.

Since the features are defined in different units, they were all normalized to unit variances based on the values calculated to describe the ligands in the training set. This allowed their variance–covariance matrix to become a convenient correlation matrix with its diagonal elements equal to 1 and the absolute values of the off-diagonal elements being less than 1. Initial weights for the combination of the features were set according to the extent of the variance explained by each feature applied to the training set, calculated according to an empirical equation,
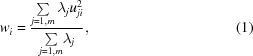
where *w_i_* is the calculated weight for the feature, *m* are the five highest value eigenvalues, λ*_j_* is the *j*th eigenvalue of the correlation matrix and *u* is the corresponding eigenvector. The weights were subsequently trained using the cross-entropy method (Rubinstein & Kroese, 2004 ▶) to maximize the rank of the correct ligand amongst the 82 candidates given the features calculated from the data in the training set. The best sparse grid was taken to be that with the lowest nearest-neighbour root-mean-square deviation (NNRMSD) to the correct ligand calculated as described in §[Sec sec3.1]3.1.

### 2.6. Small-molecule alignment and real-space refinement   

As well as for training, a conformationally flexible alignment of the ligand to the pseudo-atomic sparse grids was required following shape matching in order to place the identified ligand appropriately into the map for subsequent real-space refinement and ranking based on real-space correlation coefficients. The standalone software *ligalign* was developed in order to minimize nearest-neighbour distances between the ligand and the sparse grid.

The stereochemistry of the ligand is automatically calculated from the coordinates as described previously (Langer *et al.*, 2012 ▶). The three principal axes of the ligand (in an arbitrary conformation) are aligned with those of the grid in all four possible combinations (+*x* +*y* +*z*, −*x* −*y* +*z*, +*x* −*y* −*z* and −*x* +*y* −*z*) and each is considered independently. Alignment is achieved through rotation around the bonds (*i.e.* bonds that are deemed rotatable are stochastically rotated in increments of 60° to produce all possible conformers in which intra-atomic clashes do not occur) using a genetic algorithm (Whitley, 1994 ▶). The ligand coordinates are least-squares superimposed onto the nearest-neighbour sparse-grid points. For each conformer, a score is calculated according to
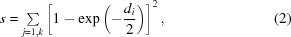
where *k* is the number of nearest-neighbour pairs and *d_i_* is the nearest-neighbour distance for each pair. Such an objective function was chosen so that in cases in which an atom of one model had two neighbours in the other, the shift of the first model is biased towards a match of one atom pair while leaving the second neighbour ‘unpaired’. In each cycle of the algorithm, the rotations associated with the best scoring overlays are crossed over, while refinement of the conformation is achieved by shaking the results of these crossovers by a maximum of ±10°.

In essence, the best conformation identified during the shape-based search through the database is superseded by *ligalign*, providing a closer match that may not be possible (or accurate enough) based on the discrete conformations in the database. *ligalign* can be seen as a preliminary real-space refinement with rotations around bonds to provide a better match to the grid that models the density rather than the density per se.

Following placement of the identified ligand onto the sparse grid, real-space refinement is applied as described previously (Langer *et al.*, 2012 ▶). This step highlights ligands matching the actual density rather than merely the sparse grids. The real-space correlation coefficient is calculated for the ligand region of the density map.

## 3. Results and discussion   

### 3.1. Matching ligands to sparse-grid substructures in the training set   

The preparation of ‘sparse-grid’ structures to indicate potential candidate atom positions within electron density was described as long ago as 1974 (Koch, 1974 ▶; Main & Hull, 1978 ▶; Isaacs & Agarwal, 1985 ▶), and has been used by Zwart *et al.* (2004 ▶) and elaborated upon by Langer *et al.* (2012 ▶) to model ligand structures. Crucially, sparse-grid construction was founded upon knowledge of the structure to be built and therefore the number of free atoms to be placed (or, in other words, the size of the sparse-grid cluster). The likely limits of electron density in space (*i.e.* the contour level at which the map should be used) and the likelihood that contiguous density at low thresholds might belong to either an adjacent ligand or solvent or otherwise be spurious were much clearer than in the current instance, in which the nature of the ligand and thus its size, shape and conformation were all to be found.

Using the methods described above and depicted in Fig. 2 ▶, we could produce pseudo-atomic representations of the density that for 93% of the cases in the training set were within 1.0 Å nearest-neighbour root-mean-square deviation (NNRMSD) of the actual ligand.

The grid substructure with the lowest NNRMSD to the true ligand resulted from the first, second and third thresholds of the density value (see Fig. 2 ▶ *c*) in almost equal numbers of cases. The fifth grid was the best in only a single case.

The ratio of the number of atoms in the ligand to the number of selected grid points kept was quite variable, fluctuating between 0.25 and 1.7; the distribution resembled a Gaussian with a mean of 0.8 and a standard deviation of 0.3. We note that here we do not construct the ligand from the sparse grid as in the label-swapping approach (Zwart *et al.*, 2004 ▶). The sparse grid only contains the number of free atoms that permit the use of shape descriptors. As the NNRMSD values demonstrated, the overall shape of the grid substructures tended to match the ligands well, and as the majority of the features used focused on the overall shape of the body, it was anticipated that differences in the numbers between the entities would be overcome by application of the features.

The concept of grid thinning based on subtle changes in the values of electron density is very similar to that of the fragmentation tree introduced by Langer *et al.* (2012 ▶), where characteristic breaks were observed in plots of density-cluster volumes against isocontour sigma thresholds when density that was contiguous with adjacent molecules fragments between the different molecular entities. In the case described here, atomic locations are used rather than density volumes, enabling the more accurate thinning of extraneous free-atom points based on distance, as shown in Figs. 2 ▶(*b*) and 2 ▶(*d*).

### 3.2. Dependence on data resolution, ligand size and conformations in the training set   

**Figure 3:**
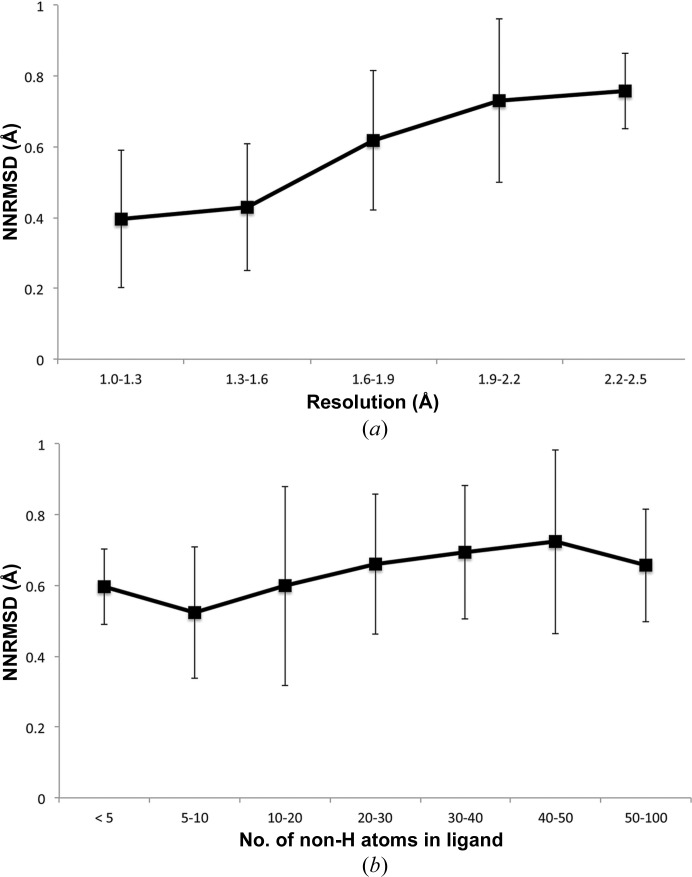
The NNRMSD differences between the sparse grids calculated for the training set and the ligand coordinates deposited in the PDB are compared for (*a*) data for various resolutions and (*b*) ligands of different sizes. The error bars depict the standard deviation of the values across the set.

Further analysis (Fig. 3 ▶) indicated that performance was dependent on the resolution of the data, but that the NNRMSD of the grid substructures to the ligand was consistent across ligands of all sizes.

Our current method for conformation generation does not test the different puckers of ring systems. Therefore, we included multiple conformations of some ligands in the database, as indicated in Table 2 ▶. The database for all tests contained 96 different molecular entities representing 82 distinct ligands, each in up to 200 conformations.

### 3.3. Performance of the feature comparisons   

Weighting of the features using the training set as described above permitted the selection of the correct compound as the top-ranked entity in 32% of the cases, without real-space refinement and the use of density correlation coefficient as an additional filtering criterion. We noted that the correct ligand was identified in the top ten following feature-based ranking in 86% of the cases and in the top 20 in 94% of the cases. We decided to pass the top 20 ranked ligands to the final real-space refinement step.

As mentioned in the Introduction[Sec sec1], the use of mathematical features individually to match ligands to their density has met with more limited success than for protein or nucleotide modelling. As ligands are much more variable chemically and conformationally relative to macromolecules and their fragments, it must be assumed that single features capturing individual aspects of a ligand or density shape are insufficient for the purpose of conformation-dependent ligand identification. Based on these results, we concluded that a combination of features describing such shapes more thoroughly should be used.

Notwithstanding the discussion in the previous paragraph, analysis of the detected weights, based on (3), indicated that features based on interatomic distances were especially suitable for the task of matching ligands to their sparse grids. The third-order moment invariants also contributed to the matching procedure to a reasonable extent, as did the features based on interatomic connectivity. The latter are conformation-invariant descriptors and thus complemented the conformation-variant features well. Notably, the three principal components of the ellipsoid about a ligand, taken as features, contributed only 0.1% of the overall contribution of the 22 features in Table 1 ▶. This highlights the importance of the third-order and higher-order features.
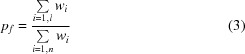
In (3), the discriminatory power, *p_f_*, of a particular category of features is calculated as the sum of all *l* weights of these features divided by the sum of weights for all 22 (*n*) features.

### 3.4. Dependence on the resolution of the data and the size of the ligand in the evaluation set   

**Figure 4:**
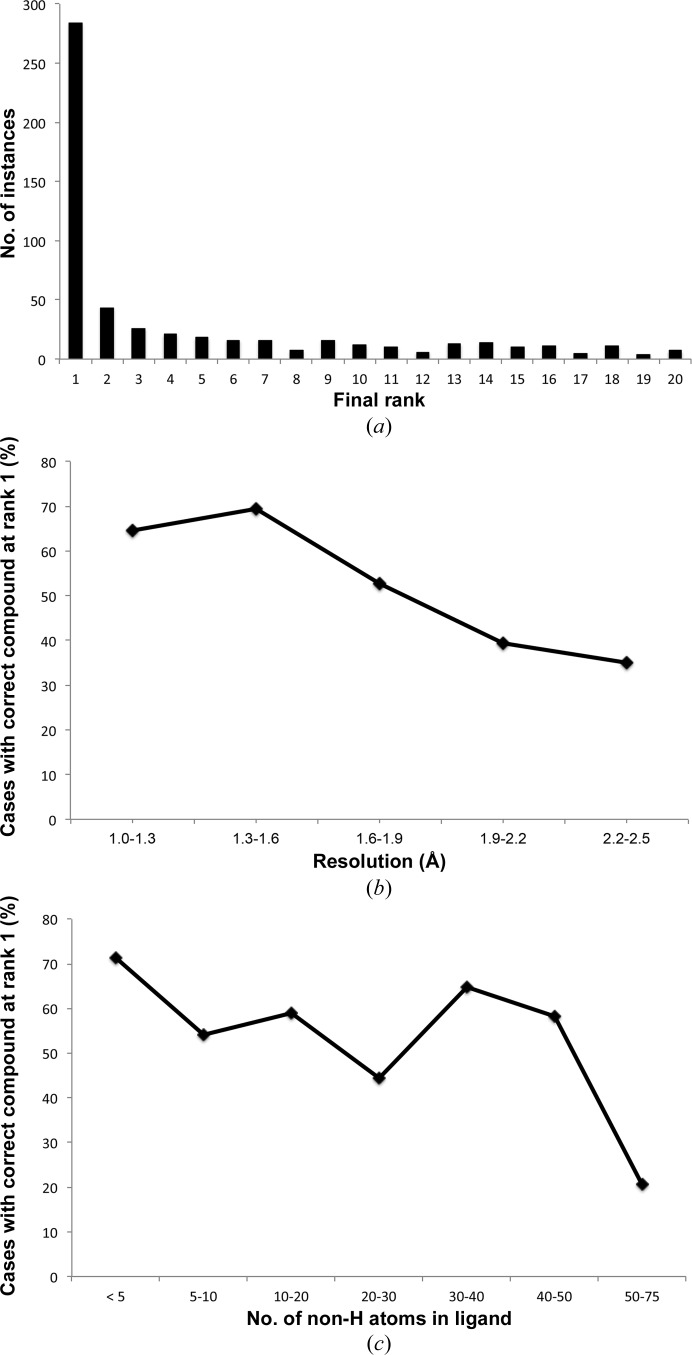
(*a*) Final ranks of the correct compound following real-space refinement and ranking by CC for the 550 compounds passing through feature-based ligand selection. (*b*) Performance with data at various resolutions amongst those ligands passed to the final real-space refinement step. (*c*) Performance with ligands of different sizes amongst those ligands passed to the final real-space refinement step.

Application of the method in its entirety to the large evaluation set of experimental data indicated that feature matching alone could identify the ligand in the top 20 ranked compounds in 61% of the cases. Real-space refinement of these 20 candidates superimposed onto the grid and re-ranking by CC placed the correct compound at the top rank in 31% of cases. As shown in Fig. 4 ▶(*a*), the correct compound was consistently ranked highly. Given that the method is looking for the correct ligand in the correct conformation with low NNRMSD, we conclude that if an appropriate sparse grid is prepared such that identification by feature comparison is possible, then application of the *ligalign* procedure followed by real-space refinement and ranking by CC is very efficient.

Further emphasizing this point was the fact that performance was very dependent on the resolution of the data, as was the case for sparse-grid construction. As highlighted in Fig. 4 ▶(*b*), approximately 70% of ligands could be accurately identified at the highest rank with data between 1.0 and 1.6 Å resolution when the compound was passed to the final refinement step. The majority of compounds are still recognized at resolutions better than 2.0 Å, but performance decreases at poorer resolutions. The reasons for this are likely founded in the free-atom-based approach whereby individual atoms and particularly the gaps between them must be identified in order to permit accurate thinning of the clusters based on interatomic distances. The procedure still shows utility with data of up to 2.5 Å resolution.

Performance is less influenced by the size of the ligands to be fitted, as highlighted in Fig. 4 ▶(*c*). Indeed, it is likely to be the composition of the ligand and whether its typical density is unique in shape that influences performance most significantly, as noted previously by Terwilliger *et al.* (2007 ▶).

### 3.5. Software implementation   

The developed technologies have been implemented in the *ARP*/*wARP* 7.4 package for crystallographic model building that was co-released in October 2013 with *CCP*4 v.6.4.0. Considering all ligand conformations, the final database for use in the software contains almost 10 000 molecular entities. Ligand identification can be accomplished intuitively through simple selection of an electron-density cluster in the graphical user interface *ArpNavigator* (Langer *et al.*, 2013 ▶) and invocation of the analysis by mouse click. The procedure is quick to execute on account of the use of pre-calculated numerical features for the ligand database. When run on a single core of a desktop workstation, the average execution time is approximately 2.5 min. Following execution, the top-ranked compound is modelled within the density. Compounds that cluster with this ligand, as described in the methods section[Sec sec2], are also output (for example, having attributed a particular electron-density cluster to a sulfate ion, a phosphate ion is offered as an alternative solution), enabling consideration of the most appropriate ligands based on the likely crystal contents. Thus, while the screening database only includes 82 ligands, the software can aid in the identification of up to 140 different compounds. The list of compounds screened is provided in Table 2 ▶.

## 4. Conclusions   

We have demonstrated that through the application of density and distance constraints to a densely packed area representing a particular cluster of difference electron density, pseudo-atomic sparse-grid structures can be obtained that closely resemble the structures of the ligands responsible for such density. Furthermore, feature-based comparisons of the sparse grids to a variety of ligand conformations can reliably point to the correct ligand. Real-space refinement of the ligands following their placement onto the grids provides a finer means of ligand discrimination. Both sparse-grid construction and ligand real-space refinement are dependent on the resolution of the X-ray data; the majority of compounds can be recognized at resolutions of better than 2.0 Å, but performance decreases thereafter.

Our analysis indicates that the ligands identified almost always fit the density blob well no matter whether they are actually correct or not. The user could therefore examine whether lower-ranked compounds might be more appropriate in any particular instance. We have found that identification errors typically arise from inaccuracies in grid preparation; these in turn often result from difficulties in identifying the boundary of a given cluster and placement of free atoms into density attributable to other ligands or metal ions. We intend to work on improving this aspect of the method in the future.

We note that although it is the combination of different shape features that is most important, the result of this combination and the estimation of the relative ‘power’ of individual groups of features depend on the objective function chosen. Here, we trained the weights for feature combination so that the rank of the correct ligand is maximized. Clearly, there are many ways in which this can be accomplished and this could be the subject of future research.

Going forward, a number of other advances can be made to the presented methodology in order to improve its accuracy and/or efficiency. Re-parameterization of sparse-grid construction and future research in improved determination of density-blob boundaries might perhaps be warranted when applied to data at lower resolutions. The addition of other features for ligand–grid comparisons might also improve recognition and as long as features can be quickly calculated and compared their inclusion in the method could be considered. It could be also worthwhile passing more ligands on to final refinement than the current 20, and the establishment of a supplementary protocol with a longer running time may be considered.

The inclusion of data derived from the protein and the consideration of protein–ligand contacts would be likely to have a significant impact on performance. This could be achieved in diverse ways, whether by inclusion of a physics-based scoring function that accounts for such interactions (Diller *et al.*, 1999 ▶) or by using ligand-binding templates in the protein (Liu & Altman, 2011 ▶). In either instance, it is likely that ligands that can take on similar shapes could be distinguished based on the relative strengths of contact formation and electrostatic clashes. However, it may not be straightforward to pre-compute a database for all possible protein–ligand interactions.

Further advances could be obtained by reducing the number of compounds to be considered. Rather than thinning the search database stringently, it would be more advantageous to only include those ligands that are feasible based on the conditions in which the crystals are grown. It is our intention to include an interface for user selection of the buffers, crystallization reagents and protein-expression systems used for a sample under analysis in the future; this would permit the population of a system-dependent database that could be added to manually prior to screening. An analogous pre-selection of the database constituents could be obtained from analysing the protein sequence and structure and extracting a data set of ligands that bind to similar proteins or binding sites. The *LigSearch* method (de Beer *et al.*, 2013 ▶) is of interest in this regard.

The methodology for ligand identification introduced here has great potential as an important step towards possible automated model building to full completion. Thereby, protein, ligands and solvent could all be modelled successively following provision of just crystallographic data and a protein sequence as input, all without any user intervention.

## Figures and Tables

**Table 1:** The 22 features used to compare the sparse-grid density representation with the set of ligands in multiple conformations

Feature type	No. of such features	Reference (where appropriate)
Third-order moment invariants	11	Lo & Don (1989 ▶); Hattne & Lamzin (2008 ▶)
Chirality index	1	Hattne & Lamzin (2011 ▶)
Features based on interatomic distances	2	Crippen & Havel (1988 ▶)
Features based on interatomic connectivity	4	Burden (1989 ▶, 1997 ▶)
Central moments of the Euclidean distances of the atomic coordinates	3	Tabachnick & Fidell (1996 ▶)
No. of atoms	1	

**Table 2:** The ligands used for training purposes, listed by PDB three-letter code with the corresponding common ligand name (either the drug name or the compound name commonly used in the literature) Those with an asterisk next to their code are screened in at least two different pucker conformations.

Ligand three-letter code	Ligand common name
017	Darunavir
1PE	Pentaethylene glycol
2GP	Guanosine 2-monophosphate
2PE	Nonaethylene glycol
5GP*	Guanosine 5-monophosphate
A3P*	Adenosine 3′,5′-diphosphate
ACO*	Acetyl coenzyme A
ADE	Adenine
ADN	Adenosine
ADP	Adenosine 5′-diphosphate
AKG	2-Oxoglutaric acid
AMP	Adenosine monophosphate
ATP*	Adenosine 5′-triphosphate
B3P	2-[3-(2-Hydroxy-1,1-dihydroxymethyl-ethylamino)-propylamino]-2-hydroxymethyl-propane-1,3-diol
BCL	Bacteriochlorophyll A
BTB	Bis-tris buffer
BTN	Biotin
C2E*	Cyclic diguanosine monophosphate
CAM	Camphor
CDL	Cardiolipin
CHD	Cholic acid
CIT	Citric acid
CLA	Chlorophyll A
CMP	Adenosine 3′,5′-cyclic monophosphate
COA	Coenzyme A
CXS	3-Cyclohexyl-1-propylsulfonic acid
CYC	Phycocyanobilin
DIO	1,4-Diethylene dioxide
DTT	1,4-Dithiothreitol
EPE	HEPES
F3S	Fe_3_–S_4_ cluster
FAD*	Flavin-adenine dinucleotide
FMN*	Flavin mononucleotide
FPP	Farnesyl diphosphate
GOL	Glycerol
GSH	Glutathione
H4B	5,6,7,8-Tetrahydrobiopterin
HC4	*para*-Coumaric acid
HEA*	Haem A
HED	2-Hydroxyethyl disulfide
HEM	Haem
IMD	Imidazole
IPH	Phenol
LDA	Lauryl dimethylamine-*N*-oxide
MES	2-(*N*-Morpholino)ethanesulfonic acid
MLI	Malonate ion
MLT	D-Malate
MPD	(4*S*)-2-Methyl-2,4-pentanediol
MTE	Phosphonic acid mono-(2-amino-5,6-dimercapto-4-oxo-3,7,8A,9,10,10A-hexahydro-4H-8-oxa-1,3,9,10-tetraaza-anthracen-7-ylmethyl)ester
MYR	Myristic acid
NAD*	Nicotinamide adenine dinucleotide
NAP*	Nicotinamide adenine dinucleotide phosphate
NCO	Cobalt hexammine(III)
NHE	2-(*N*-Cyclohexylamino)ethanesulfonic acid
OLA	Oleic acid
ORO	Orotic acid
P6G	Hexaethylene glycol
PEG	Di(hydroxyethyl)ether
PEP	Phosphoenolpyruvate
PG4	Tetraethylene glycol
PGA	2-Phosphoglycolic acid
PGO	*S*-1,2-Propanediol
PHQ	Benzyl chlorocarbonate
PLM	Palmitic acid
PLP	Pyridoxal-5′-phosphate
POP	Pyrophosphate^2−^
PYR	Pyruvic acid
RET	Retinal
SAM*	*S*-Adenosylmethionine
SF4	Iron–sulfur cluster
SIA	*O*-Sialic acid
SO4	Sulfate ion
SPO	Spheroidene
STU*	Staurosporine
TAM	Tris(hydroxyethyl)aminomethane
THP	Thymidine 3′,5′-diphosphate
TLA	L-(+)-Tartaric acid
TPP	Thiamine diphosphate
TRS	Tris buffer
TYD	Thymidine 5′-diphosphate
U10	Coenzyme Q10
UPG	Uridine 5′-diphosphate-glucose
